# Late-Onset Immune-Mediated Hepatotoxicity Induced by the Immune Checkpoint Inhibitor Pembrolizumab in Lung Cancer: A Case Report

**DOI:** 10.7759/cureus.95961

**Published:** 2025-11-02

**Authors:** Jacobo Echeverri-Hoyos, Eduardo Tuta-Quintero, Jaime A Echeverri Franco, Nicole Bonilla, Carolina Rojas-Londoño

**Affiliations:** 1 School of Medicine, Institución Universitaria Visión de las Américas, Pereira, COL; 2 Internal Medicine, Universidad de La Sabana, Chía, COL; 3 Pulmonology, Clínica de Alta Tecnología Oncólogos del Occidente, Pereira, COL; 4 Internal Medicine, Universidad de La Sabana, Bogotá, COL; 5 Internal Medicine, Universidad CES, Medellín, COL

**Keywords:** adverse event, hepatotoxicity, liver, lung cancer, pembrolizumab

## Abstract

Lung cancer (LC) remains the leading cause of cancer-related death worldwide. The introduction of immune checkpoint inhibitors, such as pembrolizumab, has improved survival in selected patients. However, these therapies may cause immune-mediated adverse events, including hepatotoxicity, which poses a diagnostic challenge due to its low incidence and nonspecific presentation. We report a case of a patient with LC who developed persistent elevation of liver enzymes after 17 cycles of pembrolizumab, with no infectious or metabolic abnormalities, consistent with immune-mediated drug-induced liver injury. This case underscores the importance of continuous liver function monitoring throughout immunotherapy, even after long-term exposure, to enable early recognition and management of delayed immune-related hepatotoxicity.

## Introduction

Lung cancer (LC) is the leading cause of cancer-related death worldwide, accounting for 12.4% of all cancer diagnoses in 2022, with an estimated 20 million new cancer cases and 9.7 million deaths [1,2]. Therapeutic approaches for LC have evolved significantly, incorporating strategies that target specific genetic alterations and the immune system [3,4]. Among these, immune checkpoint inhibitors, such as programmed cell death-1 (PD-1) receptor blockers, have improved prognosis in selected patient subgroups [3,5].

Pembrolizumab is a monoclonal IgG4 antibody that inhibits PD-1 on T cells, enhancing the antitumor immune response [5,6]. However, this mechanism can also trigger dysregulated immune activation, resulting in immune-mediated adverse effects that may affect multiple organs, including the liver [5-7]. Although hepatic toxicity from immune checkpoint inhibitors is uncommon, it can be severe and potentially life-threatening if not promptly recognized [3,5,7].

Drug-induced liver injury (DILI) is generally defined by elevated liver enzymes per DILIN (Drug-Induced Liver Injury Network) criteria (e.g., alanine aminotransferase (ALT) ≥5× ULN, alkaline phosphatase (ALP) ≥2× ULN, or total bilirubin ≥2.5 mg/dL) [8-12]. In patients receiving pembrolizumab, hepatotoxicity is associated with worse prognosis and increased risk of complications, and its low incidence makes diagnosis and management particularly challenging [1,2,7]. We present a case of delayed-onset liver toxicity after multiple cycles of pembrolizumab in a patient with lung cancer, highlighting the clinical relevance of vigilant monitoring and early recognition of immune-mediated hepatotoxicity during long-term immunotherapy.

## Case presentation

**Figure 1:**
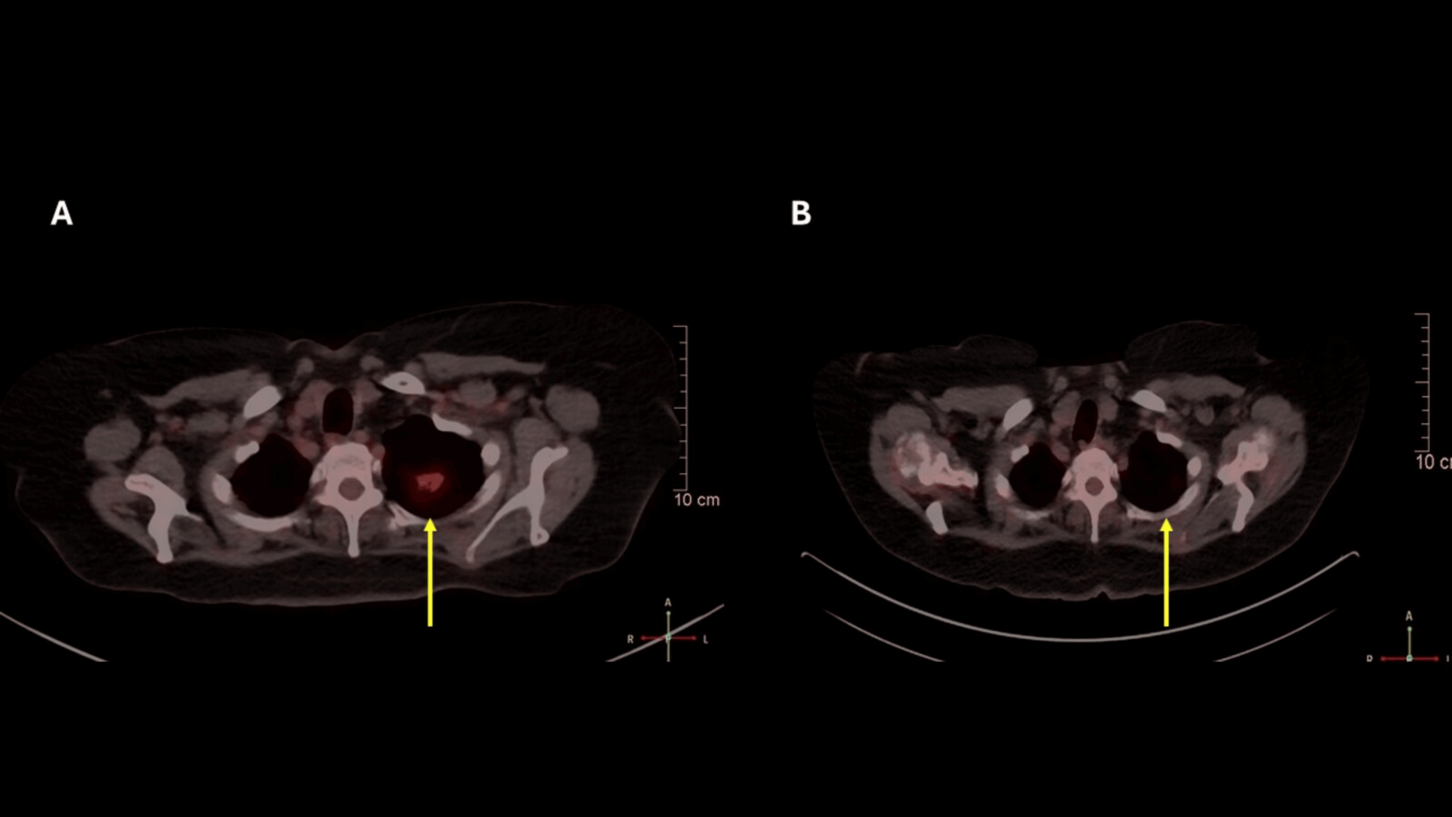
Positron Emission Tomography Before Surgery and After Pembrolizumab Therapy. A: A hypermetabolic nodular lesion is observed (maximum standardized uptake value: 4.6), with spiculated margins, pleural contact, and an internal air bronchogram, measuring 33 × 29 × 22 millimeters, located in the left posterior apical segment. B: No hypermetabolic lesions are detected on the six-month oncologic follow-up positron emission tomography-computed tomography after pembrolizumab therapy.

A 75-year-old woman with a history of hypothyroidism and previously treated poorly differentiated, high-grade squamous cell carcinoma of the right upper lobe (stage T2aN0M0) underwent lobectomy and adjuvant gemcitabine-cisplatin chemotherapy 10 years earlier and had been on annual surveillance with chest computed tomography (CT). The most recent CT scan revealed a 33 × 29 × 22 mm lesion in the posterior apical segment of the left upper lobe (Figure 1A). Positron emission tomography (PET) showed a poorly defined, hypermetabolic lesion with pleural extension (maximum standardized uptake value (SUVmax) 4.6). Endoscopic ultrasound-guided biopsy confirmed adenocarcinoma with lepidic and micropapillary components, without mediastinal nodal involvement (stations 4L, 7, 11L, and 11R).

The patient underwent a left upper lobe segmentectomy with systematic mediastinal lymph node sampling, including interlobar (station 11) and supracarinal (station 7) nodes. Histopathology confirmed invasive micropapillary adenocarcinoma, grade 3, with spread through air spaces (STAS) and a 0.4 cm surgical margin. Programmed death-ligand 1 (PD-L1) expression was <1% (SP263, Ventana), and next-generation sequencing (NGS) revealed no actionable mutations in EGFR, ALK, KRAS, BRAF, MET, PIK3CA, RET, ROS1, or NTRK1-3 genes. These findings were consistent with a second metachronous primary tumor (pathologic stage pT2aN0M0).

**Figure 2:**
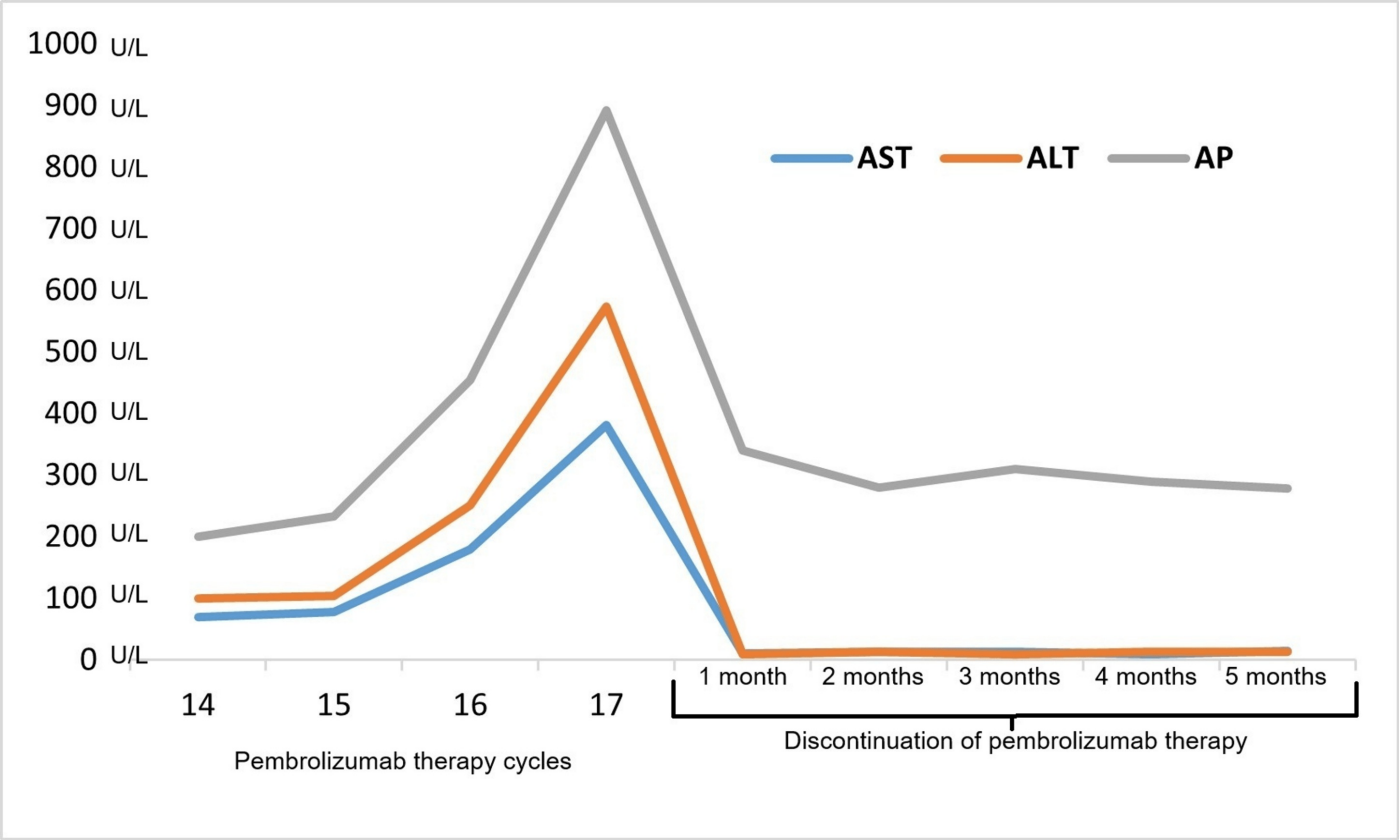
Temporal changes in liver enzymes and alkaline phosphatase in relation to the number of pembrolizumab doses U/L: International Units per liter; ALT, alanine aminotransferase; AST, aspartate aminotransferase; AP, alkaline phosphatase

Given the high-risk histological features, adjuvant pembrolizumab (200 mg every 21 days) was initiated under an institutional, off-label, compassionate-use protocol. After 17 cycles, the patient developed persistent elevations in ALT, aspartate aminotransferase (AST), and ALP (Figure 2), leading to treatment discontinuation. Coagulation parameters, total bilirubin, and albumin remained within normal ranges; viral hepatitis and other causes of DILI were excluded. Corticosteroids were not required, and liver enzyme levels progressively improved following the interruption of pembrolizumab, with complete normalization by the fifth month of follow-up. At the six-month oncologic follow-up, PET-CT demonstrated a complete metabolic response with no evidence of residual disease (Figure 1B).

## Discussion

This case report is relevant, as it documents an adverse reaction that led to treatment discontinuation after more than a year of adequate tolerance, highlighting the need for continuous liver monitoring throughout the entire course of immunotherapy, even during late stages. It also provides useful evidence for the diagnostic and therapeutic approach when evaluating enzymatic elevations in this clinical context. The isolated and sustained elevation of liver enzymes after multiple cycles of pembrolizumab, in the absence of coagulation abnormalities, bilirubin changes, or infectious markers, suggests possible immune-mediated hepatotoxicity [10-12]. Although hepatotoxicity induced by immune checkpoint inhibitors is a recognized complication, its presentation can be delayed, subtle, and easily attributed to other etiologies, particularly in patients with complex oncologic histories and multiple prior lines of therapy [7,8,10-16].

Immune-related hepatotoxicity associated with immune checkpoint inhibitors can present with heterogeneous clinical and biochemical patterns, often mimicking other causes of liver injury [12-22]. In our review of published cases, we observed a predominance of hepatocellular or mixed enzyme elevations, frequently occurring after only a few cycles of pembrolizumab [12-22]. Diagnostic confirmation was typically based on the temporal association with immunotherapy, exclusion of alternative causes, and, in some instances, histological findings consistent with immune-mediated injury [12-22]. These observations underscore the diagnostic challenges and highlight the need for early suspicion in patients receiving immunotherapy. Table 1 details the clinical features, oncologic diagnosis and stage, immunotherapy regimen (cycles), key laboratory findings, and criteria supporting the diagnosis of DILI in these reported cases [12-22].

**Table 1:** Case reports evaluating hepatic involvement during pembrolizumab therapy DILI: drug-induced liver injury; ALT: alanine aminotransferase; AST: aspartate aminotransferase; PD-L1: programmed death-ligand 1; IV: intravenous; q: every; NSCLC: non-small cell lung cancer; mg: milligrams; wk: weeks Sources: [12-22]

Age/Sex	Oncologic Diagnosis	Immunotherapy Treatment / Cycles	Laboratory Findings	DILI Criteria
65 years, Female [12]	Stage IVB pulmonary adenocarcinoma (cT4N3M1c).	Pembrolizumab 200 mg IV every 3 weeks (4 cycles).	Elevated ALT, AST, alkaline phosphatase, and gamma-glutamyl transpeptidase.	Clear temporal relationship, negative serology, and histological confirmation.
68 years, Male [13]	Advanced non-small cell lung cancer (NSCLC), high PD-L1 (>50%).	Pembrolizumab.	Elevated ALT, AST, and alkaline phosphatase.	Clinical course, absence of alternative causes, and response to immunosuppressive therapy.
79 years, Male [14]	Right pulmonary adenocarcinoma cT4N3M0, stage IIIC, PD-L1 100%.	Pembrolizumab (1 cycle).	Elevated ALT and AST.	Immediate temporal relationship, no alternative causes.
73 years, Male [15]	Metastatic squamous cell carcinoma of the head and neck.	Pembrolizumab (1 cycle).	Elevated ALT, AST, and alkaline phosphatase.	Temporal relationship, no alternative causes, resolution with corticosteroids.
86 years, Male [16]	Metastatic gastric cáncer.	Pembrolizumab 100 mg every 2 weeks (5 cycles).	Elevated total and direct bilirubin, gamma-glutamyl transpeptidase, and alkaline phosphatase. AST and ALT are within normal limits.	Immune-related cholestatic hepatitis (no obstruction, no tumor progression, other causes ruled out).
67 years, Male [17]	Metastatic melanoma.	Pembrolizumab.	Elevated alkaline phosphatase, gamma-glutamyl transferase, AST, ALT, and total bilirubin.	Temporal relationship, no reported obstructive cause, and histological confirmation.
56 years, Female [18]	Stage IV pulmonary adenocarcinoma with metastasis and associated stage T3N0M0 squamous cell carcinoma of the tonsil.	Pembrolizumab 200 mg IV every 3 weeks + radiotherapy (3 cycles).	Elevated ALT, AST, alkaline phosphatase, and gamma-glutamyl transpeptidase.	Exclusion of other causes and histological confirmation.
73 years, Male [19]	Metastatic melanoma with lung metastases and associated retinopathy.	Pembrolizumab (7 cycles, suspended 9 weeks prior to hepatitis onset).	Elevated ALT, AST, alkaline phosphatase, and bilirubin.	Late-onset hepatitis, steroid-refractory.
58 years, Male [20]	p16+ oropharyngeal squamous cell carcinoma T4N2M0 with lung metastases.	Pembrolizumab + Cisplatin + 5-fluorouracil (5 cycles), then pembrolizumab monotherapy.	Elevated ALT and AST.	Temporal relationship, alternative causes ruled out, steroid-refractory.
48 years, Female [21]	Oropharyngeal squamous cell carcinoma cT2N3bM0 with jugular chain recurrence.	Pembrolizumab + Cisplatin + 5-fluorouracil (11 cycles).	Elevated ALT and AST.	Positive lymphocyte transformation test only for pembrolizumab, resolved without immunosuppressive treatment.
80 years, Male [22]	Recurrent urothelial carcinoma of the renal pelvis.	Pembrolizumab.	Elevated ALT, AST, alkaline phosphatase, gamma-glutamyl transpeptidase, and amylase.	Liver biopsy showing infiltration by lymphocytes, neutrophils, and eosinophils.

The pathophysiology of immune checkpoint inhibitor-induced hepatotoxicity is not fully understood but is thought to involve dysregulated immune activation [15-20]. Proposed mechanisms include T-cell-mediated cytotoxicity against hepatocytes, imbalance of pro- and anti-inflammatory cytokines, and loss of immune tolerance, which together can trigger liver inflammation even after prolonged treatment [14-18]. Recognizing these underlying processes helps contextualize the delayed onset observed in this case and may inform strategies for early detection and management. The diagnosis of DILI represents a considerable clinical challenge, as it can mimic a wide range of acute and chronic hepatobiliary diseases [8,9,12-14]. In this case, given the persistent elevation of transaminases after multiple cycles of pembrolizumab, a systematic approach was required to rule out other potential causes of liver dysfunction.

In this case, the patient developed a progressive increase in ALT and AST after receiving 15 cycles of pembrolizumab, without associated clinical manifestations or evidence of severe hepatic dysfunction. Although hepatotoxicity induced by immune checkpoint inhibitors typically presents within the first six months of treatment, this delayed onset underscores that immune-mediated complications can arise even after prolonged and seemingly well-tolerated exposure. In response to this biochemical finding, several potential causes, including viral infection, tumor infiltration, ischemic injury, and drug-induced toxicity, were considered and ruled out through complementary studies. The progressive normalization of ALT and AST after discontinuation of the immunotherapeutic agent supports the diagnosis of probable immune-mediated hepatitis [12-14]. This outcome highlights the importance of maintaining close monitoring throughout the entire course of treatment, even in asymptomatic patients with previously good tolerance.

The definition of DILI in this case was based on the criteria established by the DILIN [8,9]. After the sixteenth administration of pembrolizumab, the patient developed a sustained increase in ALT, AST, and alkaline phosphatase, without abnormalities in total bilirubin, albumin, or coagulation times (prothrombin time (PT), activated partial thromboplastin time (aPTT)) [8-12]. This biochemical profile was consistent with a mixed (hepatocellular-cholestatic) pattern of liver injury attributable to immunotherapy, leading to treatment discontinuation at cycle 17. The progressive normalization of liver enzymes after discontinuation of the drug, along with the exclusion of other causes, including viral infections, supported the diagnosis of pembrolizumab-induced immune-mediated DILI [12-15].

The differential diagnosis included infectious etiologies, such as viral hepatitis B and C, metabolic and autoimmune hepatopathies, including ischemic hepatitis, autoimmune hepatitis, hemochromatosis, and Wilson’s disease, as well as malignant causes such as hepatocellular carcinoma or pancreatobiliary neoplasms [12-18]. However, the absence of clinical or paraclinical evidence supporting any of these alternatives, together with the normalization of liver enzymes after the discontinuation of pembrolizumab, supported the diagnosis of a probable Grade 2 immune-mediated DILI according to CTCAE v5.0, consistent with a hepatocellular pattern of injury secondary to immune checkpoint inhibitor exposure [8,9].

A literature review showed that most reported cases corresponded to elderly patients (range: 48-86 years), with a predominance of males [12-22]. Underlying oncologic conditions primarily included pulmonary adenocarcinoma, metastatic melanoma, and squamous cell carcinomas of the head and neck [12-22]. In all reports, a causal relationship between pembrolizumab use and the hepatic event was established based on the temporal association, exclusion of other causes, and, in some cases, histological confirmation. The most common laboratory findings were elevations in aminotransferases, alkaline phosphatase, and GGT, with some patients also presenting hyperbilirubinemia or a cholestatic pattern. The reports highlight diverse patterns of presentation, ranging from early-onset immune-mediated hepatitis after one or a few treatment cycles to late-onset cases occurring after drug discontinuation. Several patients demonstrated clinical resolution following corticosteroid therapy, although cases of poor response or refractoriness were also reported [12-22]. One case showed spontaneous resolution without the need for immunosuppression, and another was confirmed via lymphocyte transformation testing [12-22].

The decision to initiate adjuvant pembrolizumab in this case of PD-L1-negative early-stage NSCLC was supported by emerging evidence from the phase III PEARLS/KEYNOTE-091 trial, which demonstrated a disease-free survival benefit in patients with complete resection in stages IB-IIIA, regardless of PD-L1 expression [23]. Recent commentary from regulatory agencies and experts has interpreted these results as a potential extension of pembrolizumab's applicability beyond PD-L1-positive tumors, particularly in patients at higher risk of recurrence [23,24]. Furthermore, high-risk histologic features, such as micropapillary architecture, a recognized risk factor for early relapse, provide additional biological rationale for considering immunotherapy in carefully selected cases [24,25]. The use of pembrolizumab in our patient was strictly performed under close monitoring and informed consent from the patient.

## Conclusions

This case illustrates the potential for late-onset hepatic toxicity during adjuvant immunotherapy, even after multiple well-tolerated cycles. It emphasizes the importance of continuous and careful monitoring of liver enzymes throughout treatment, as immune-mediated adverse events can present in a delayed and subtle manner. Moreover, it highlights the need for personalized follow-up strategies and timely therapeutic adjustments, contributing to a better understanding of immune-related hepatotoxicity and guiding future monitoring protocols to optimize patient safety while preserving the efficacy of immunotherapy.
